# Rootstock–Scion Interaction Affects Cadmium Accumulation and Tolerance of *Malus*

**DOI:** 10.3389/fpls.2020.01264

**Published:** 2020-08-14

**Authors:** Jiali He, Jiangtao Zhou, Huixue Wan, Xiaolei Zhuang, Huifeng Li, Sijun Qin, Deguo Lyu

**Affiliations:** ^1^ College of Horticulture, Shenyang Agricultural University, Shenyang, China; ^2^ Key Lab of Fruit Quality Development and Regulation of Liaoning Province, Shenyang Agricultural University, Shenyang, China; ^3^ Research Institute of Pomology, Chinese Academy of Agricultural Sciences, Xingcheng, China; ^4^ Institute of Pomology, Shandong Academy of Agricultural Sciences, Tai’an, China

**Keywords:** heavy metal, apple, rootstock, scion, detoxification, transcriptional regulation

## Abstract

To understand the roles of *Malus* rootstock, scion, and their interaction in Cd accumulation and tolerance, four scion/rootstock combinations consisting of the apple cultivars “Hanfu” (HF) and “Fuji” (FJ) grafted onto *M. baccata* (Mb) or *M. micromalus* “qingzhoulinqin” (Mm) rootstocks differing in relative Cd tolerance were exposed either to 0 µM or 50 µM CdCl_2_ for 18 d. Cd accumulation and tolerance in grafted *Malus* plants varied within rootstock, scion, and rootstock–scion interaction. Cd-induced decreases in photosynthesis, photosynthetic pigment level, and biomass were lower for HF grafted onto Mb than those for HF grafted onto Mm. Reductions in growth and photosynthetic rate were always the lowest for HF/Mb. Cd concentration, bioconcentration factor (BCF), and translocation factor (*T_f_*) were always comparatively higher in HF and FJ grafted onto rootstock Mm than in HF and FJ grafted on Mb, respectively. When HF and FJ were grafted onto the same rootstock, the root Cd concentrations were always higher in HF than FJ, whereas the shoot Cd concentrations displayed the opposite trend. The shoot Cd concentrations and *T_f_* were lower for HF/Mb than the other scion/rootstock combinations. Rootstock, scion, and rootstock–scion interaction also affected subcellular Cd distribution. Immobilization of Cd in the root cell walls may be a primary Cd mobility and toxicity reduction strategy in *Malus*. The rootstock and scion also had statistically significant influences on ROS level and antioxidant activity. Cd induced more severe oxidative stress in HF and FJ grafted onto Mm than it did in HF and FJ grafted onto Mb. Compared with FJ, HF had lower foliar O_2_^-^, root H_2_O_2_, and root and leaf MDA levels, but higher ROS-scavenging capacity. The rootstock, scion, and rootstock–scion interaction affected the mRNA transcript levels of several genes involved in Cd uptake, transport, and detoxification including *HA7*, *FRO2-like*, *NRAMP1*, *NRAMP3*, *HMA4*, *MT2*, *NAS1*, and *ABCC1*. Hence, the responses of grafted *Malu*s plants to Cd toxicity vary with rootstock, scion, and rootstock–scion interaction.

## Introduction

Cadmium (Cd) has accumulated in orchard soils as a consequence of industrial activity and agrochemical misuse and overuse (Pizzol et al., 2014; Sungur, 2016). Cd is a non-essential and highly phytotoxic element. It impairs root function, inhibits nutrient uptake and assimilation, impedes photosystem II activity, lowers carbohydrate concentrations, hinders protein synthesis, suppresses plant growth, and limits fruit yield (Lopez-Climent et al., 2011; Stachowiak et al., 2016). Cd accumulation in edible plant parts may ultimately enter the human body *via* the food chain and poses a serious threat to human health (Nawrot et al., 2006). From agronomic and food safety perspectives, then, it is important to determine how to mitigate Cd toxicity and reduce Cd uptake and accumulation in plant tissues.

Several approaches have been proposed to attenuate Cd accumulation and phytotoxicity in crops (Nawaz et al., 2016; Qian et al., 2017; Zhou et al., 2017). Grafting affects crop yield and quality and enhances plant tolerance to various stressors such as heat, drought, and heavy metals (HM) (Savvas et al., 2010; Yuan et al., 2019). Certain rootstocks prevent Cd uptake and/or transport by ion exclusion or retention. In this manner, they mitigate heavy metal phytotoxicity. Grafting eggplant and tomato scions onto *Solanum torvum* rootstocks decreased foliar Cd accumulation (Yuan et al., 2019). Grafting cucumber (*Cucumis sativus* L.) scions onto “Power” (*C. maxima* × *C. moschata*) rootstocks reduced Cd absorption and transport (Savvas et al., 2013). However, the shoots may also influence the mechanisms that control root HM uptake and accumulation by altering metabolite transport (Savvas et al., 2010). Thus, HM uptake and accumulation depend on the rootstock, the scion, and the rootstock–scion interaction (Liao et al., 2015). Nevertheless, the complex communication between rootstock and scion that regulates HM uptake has not yet been elucidated.

Plants have evolved various Cd detoxification strategies (He et al., 2015). They may bind Cd to cell walls, compartmentalize it into vacuoles, and restrict its accumulation in sensitive organelles (Luo et al., 2016). Scions can affect subcellular HM distribution in rootstock roots. When Ralls and Fuji apple cultivars were grafted onto the same *Malus hupehensis* rootstock, the former sequestered more Cu in the root cell walls than the latter (Wang et al., 2016). To the best of our knowledge, however, little is known about the effects of the rootstock, the scion, and the rootstock–scion interaction on subcellular Cd distribution in woody crops such as fruit trees.

In recent years, remarkable progress has been made on clarifying the physiological responses of crops to Cd exposure (Romero-Puertas et al., 2012; Edelstein and Ben-Hur, 2018). In certain plant species, Cd phytotoxicity is manifested by reactive oxygen species (ROS) and malonaldehyde (MDA) induction (Luo et al., 2016). Nonenzymatic antioxidants such as free proline, soluble phenolics, ascorbate (ASC), and reduced glutathione (GSH), and several antioxidant enzymes scavenge Cd-induced ROS and protect cells from oxidative damage (He et al., 2015). Both rootstock and scion influence ROS and antioxidant responses to Cd exposure. These mechanisms have been explored in herbaceous crops such as watermelon (Shirani Bidabadi et al., 2018) and tomato (Gratao et al., 2015). Therefore, scions grafted onto the appropriate rootstocks could attenuate Cd phytotoxicity by increasing antioxidant enzyme activity, raising the levels of nonenzymatic antioxidants, and decreasing ROS and lipid peroxidation (Kumar et al., 2015). Compared to herbaceous crops, the toxic effects of Cd on perennial woody plants such as fruit trees are more severe as these species have very long growth periods and, by extension, prolonged soil Cd exposure (Peryea, 2001). However, relatively little is known about the responses of ROS and antioxidants to Cd exposure in woody crops such as fruit trees.

Cd uptake, transport, and detoxification are mediated by several genes (Luo et al., 2016). Plasma membrane (PM) H^+^-ATPases furnish the proton motive force for ion transmembrane transport (Ma et al., 2014). *HA7*, *HA2.1*, and *AHA10.1* encode various H^+^-ATPases that play important roles in Cd uptake (He et al., 2015; He et al., 2020). *FRO2-like* encoding Fe(III) reductase proteins and natural resistance-associated macrophage protein 1 (NRAMP1) are located in the PM and control Cd penetration into root cell cytosols (Gao et al., 2011; Lin and Aarts, 2012). Tonoplast-localized NRAMP3 and PM-localized HM ATPase 4 (HMA4) transport Cd from the root to the shoot (Verret et al., 2004; Lin and Aarts, 2012). In the cytosol, Cd cations and phytochelatin (PC)-Cd chelates are sequestered in vacuoles by tonoplast-localized cation exchanger 4 (CAX4) and ATP-binding cassette transporter C1 (ABCC1), respectively (Mei et al., 2009; Park et al., 2012). Nicotianamine synthase 1 (NAS1) is the key enzyme regulating nicotianamine (NA) biosynthesis (Weber et al., 2004). Cd is chelated by NA and metallothionein (MT) which detoxify cytosolic Cd (Cobbett and Goldsbrough, 2002; Luo et al., 2016). Previous studies showed that the rootstock significantly affects stress-related gene expression in the scion (Jensen et al., 2003; Si et al., 2010). Hence, certain signals transported from the root to the shoot might influence Cd uptake and tolerance. Nevertheless, transcriptional regulation of the genes involved in Cd uptake, translocation, and detoxification in fruit tree scion/rootstock combinations remains largely unknown.

In a previous study, we compared Cd accumulation and tolerance among various apple rootstocks. *Malus baccata* and *M. micromalus* “qingzhoulinqin” markedly differed in terms of Cd uptake, translocation, and detoxification (Zhou et al., 2016; Zhou et al., 2017). To elucidate the physiological and molecular mechanisms by which the *Malus* rootstock, scion, and their interaction control Cd accumulation, translocation, and tolerance, we prepared graft combinations consisting of *Malus domestica* “Hanfu” (HF) and *M*. *domestica* “Fuji” (FJ) scions grafted onto either *M. baccata* (Mb) or *M. micromalus* “qingzhoulinqin” (Mm) rootstocks and exposed them either to 0 µM or 50 µM CdCl_2_ for 18 d. We hypothesized that (i) the rootstock, the scion, and their interaction influence Cd accumulation and translocation, and (ii) the rootstock–scion interaction regulates physiological acclimation and the key genes involved in Cd uptake and transport, thereby alleviating Cd toxicity. To test these hypotheses, we evaluated and compared growth characteristics, Cd accumulation and translocation, subcellular Cd distribution, oxidants, antioxidants, and the key genes regulating Cd uptake, translocation, and detoxification.

## Materials and Methods

### Plant Material and Cd Exposure

The apple cultivars “Hanfu” (HF) and “Fuji” (FJ) were grafted onto *M. baccata* (Mb) or *M. micromalus* “qingzhoulinqin” rootstocks and used in this study. Hereafter, “Hanfu” and “Fuji” grafted onto *M. baccata* are referred to as HF/Mb and FJ/Mb while “Hanfu” and “Fuji” grafted onto *M. micromalus* “qingzhoulinqin” are referred to as HF/Mm and FJ/Mm, respectively. Seeds of the *M. baccata* and *M. micromalus* “qingzhoulinqin” rootstocks were stratified in sand at 0–4°C for 60 d. The seedlings were transferred in early April 2016 to nursery plates containing substrate and cultivated for 40 d in a greenhouse under natural light and temperature conditions (26°C day/18°C night; RH = 50–60%). Seedlings with similar growth from two rootstock species were selected and transplanted in plastic pots (20 cm × 20 cm ×18 cm) containing sand. One seedling was planted per pot. Each plant was slowly chemigated every 2 d with 50 mL Hoagland solution. In April 2017, virus-free “Hanfu” and “Fuji” budwood was grafted onto Mb and Mm rootstocks that were uniform in diameter (4–5 mm). All plants were cultivated in a greenhouse under the aforementioned environmental conditions. Twelve weeks after budding, healthy and uniform grafted plants (height 35 cm) were selected and transplanted to aerated Hoagland nutrient solution (pH 6.0) that was renewed every 3 d. After 2 weeks, 36 plants similar in height and growth performance were selected from each graft combination, divided into two groups of 18 plants, and cultivated for 18 d in Hoagland solution containing either 0 µM or 50 µM CdCl_2_. Six plants per group were randomly selected for histochemical root and stem analyses. The remaining 12 plants per group were harvested after gas exchange measurements.

### Gas Exchange Measurement and Harvest

Before harvest, gas exchange parameters were measured using mature leaves (leaf plastochron index = 9–12) of each plant from four apple scion/rootstock combinations. The CO_2_ assimilation rate (*A*), stomatal conductance (*g_s_*) and transpiration rate (*E*) were measured on sunny days between 9:00 and 11:00 h with the CIRAS-2 photosynthesis system (PP Systems, USA) as described previously (Wang et al., 2013).

After gas exchange measurement, each plant was harvested *via* separation of root, rootstock stem, scion stem and leaf tissues. The roots were carefully rinsed in 20 mM EDTA disodium solution for 5 min to remove Cd^2+^ from the root surface, and then washed with deionized water. All of the harvested samples were wrapped with tinfoil after the fresh weight of samples were recorded and frozen immediately in liquid nitrogen. The frozen samples were milled to ﬁne powder in liquid nitrogen with a ball mill (MM400, Retsch, Haan, Germany) and stored at −80°C. To perform biochemical and gene expression analysis, equal amounts of powder from different tissues of four plants that received the same treatment were pooled to form a biological sample (i.e., three independent biological samples for each treatment). The fresh powder (ca. 50 mg) obtained from each tissue per plant was dried at 60°C for 72 h to determine the fresh-to-dry mass ratio, which was used to calculate the dry mass of each tissue.

### Histochemical Staining of Cd

Cd distribution was investigated in the root and scion stem tissues of apple trees using the histochemical staining method as described by He et al. (2013) with some modifications. In brief, hand sections or intact tissues from fresh samples of fine roots and scion stems were rinsed in de-ionized H_2_O. Subsequently, the samples were exposed to a staining solution (30 mg of diphenylthiocarbazone dissolved in 60 ml of acetone, 20 ml of H_2_O and seven drops of glacial acetic acid) for 90 s. After a brief rinse in deionized H_2_O, the well-stained samples with Cd-dithizone precipitates that displayed as red coloration were immediately photographed under an Eclipse E200 light microscope (Nikon, Tokyo, Japan) using a CCD camera (DS-Fi1; Nikon) connected to a computer.

### Analysis of Foliar Pigments, Cd, Bio-Concentration (BCF) and Translocation Factor (*T_f_*)

To measure contents of foliar pigments, fine powder of fresh leaves (ca. 50 mg) was extracted in 5 ml of 80% acetone for 24 h in darkness until the color disappeared completely. The contents of chlorophyll in the extracts were determined using a spectrophotometer (UV-3802, Unico Instruments Co. Ltd, Shanghai, China) as suggested by Wellburn (1994).

The fine powder (ca. 100 mg) samples obtained from the root, rootstock stem, scion stem and leaf tissues were digested in a mixture containing 7 ml concentrated HNO_3_ and 1 ml concentrated HClO_4_ at 170°C as described previously (Zhou et al., 2017). The Cd concentrations in different tissues were determined using graphite furnace atomic absorption spectrophotometry (Hitachi 180-80, Hitachi Ltd, Tokyo, Japan). Bio-concentration factor (BCF) was defined as the ratio of Cd concentration (μg g^−1^) in roots, rootstock stems, stem scions and leaves to that in the solution (μg g^−1^) (Zacchini et al., 2009). The translocation factor (*T_f_*) was defined as Cd concentration (μg g^−1^) in the aerial tissues of a plant divided by that in the roots and multiplied by 100% (Zacchini et al., 2009).

### Determination of Cd Subcellular Distribution

Root and leaf samples were separated into four subcellular fractions (cell wall fraction, organelle-rich fraction, membrane-containing fraction, and soluble fraction) using the gradient centrifugation technique according to Fu et al. (2011). Briefly, plant tissues were homogenized in 10 ml of pre-cold extraction buffer (pH 7.5) which contained 50 mM HEPES, 500 mM sucrose, 1.0 mM dithioerythritol, 5.0 mM ascorbic acid, and 1.0% w:v Polyclar AT PVPP. The homogenate was sieved through a nylon cloth (100 µm mesh size) and the residue was designated as the cell wall fraction (F I). Subsequently, the filtrate was centrifuged at 10,000 g for 30 min, and the resultant deposition was the organelle-rich fraction. The left supernatant was continued centrifuged at 100,000 g for 30 min, and the pellet retained was the membrane-containing fraction, while the supernatant was designated as the soluble fraction. All the above steps were performed at 4°C. The four fractions were evaporated at 65°C to dryness, and then digested with a mixture (7 ml concentrated HNO_3_ and 1 ml concentrated HClO_4_) at 170°C. Cd concentration in different fractions was determined by graphite furnace atomic absorption spectrophotometry (Hitachi 180-80, Hitachi Ltd, Tokyo, Japan).

### Determination of O_2_^•−^, H_2_O_2_ and MDA

The concentrations of O_2_ **^.−^** and H_2_O_2_ in the roots and leaves of plants were measured using a spectrophotometer at 530 and 410 nm, respectively, as suggested by He et al. (2011).

The concentrations of MDA in the roots and leaves were determined spectrophotometrically at 450, 532 and 600 nm, according to the previous study (Lei et al., 2007).

### Assays of Non-Enzymatic Metabolites and Antioxidative Enzyme Activities

The concentration of free proline and soluble phenolics was determined spectrophotometrically as reported previously (He et al., 2011). Ascorbate (ASC) and reduced glutathione (GSH) was measured based on the protocol described by Chen et al. (2011).

The soluble proteins in the root and leaf tissues were extracted and quantified according to Luo et al. (2008). The enzyme activities of catalase (CAT), ascorbate peroxidase (APX) and glutathione reductase (GR) were determined according to the method of Ding et al. (2017).

### Determination of the Transcript Levels of the Genes Involved in Cd Uptake and Translocation

Total RNA extraction and purification and RT-PCR were performed according to the methods of Zhou et al. (2016). Total root and leaf RNA was extracted and purified with a plant RNA extraction kit (R6827; Omega Bio-Tek, Norcross, GA, USA) and treated with DNase I (M6101; Promega, Madison, WI, USA) to eliminate genomic DNA. The concentration and quality of the extracted RNA samples were analyzed by agarose gel electrophoresis and spectrophotometry (NanoDrop 2000; Thermo Fisher Scientific, Waltham, MA, USA), respectively. The purified total RNA was used to synthesize first-strand cDNA in a PrimeScript RT reagent kit with gDNA Eraser (DRR037A; Takara, Dalian, China) according to the manufacturer’s instructions. Quantitative PCR was conducted on the genes in a reaction system containing 10 μL of 2× SYBR Green Premix Ex Taq II (DRR820A; Takara, Dalian, China), 2 μL cDNA, and 0.2 μL of 20 mM primer specifically designed for each gene (**Table S1**). The reaction was run in a CFX96 real time system (CFX96; Bio-Rad Laboratories, Hercules, CA, USA). β-Actin was the reference gene. PCR product homogeneity was confirmed with a melting curve program. PCR was conducted in triplicate for each gene. Relative mRNA expression was calculated according to the 2^−ΔΔCt^ method (Livak and Schmittgen, 2001). The expression level was set to unity for each gene in the roots or leaves of “Hanfu” (HF) grafted on *M. baccata* (Mb) rootstock not exposed to Cd. The corresponding fold changes in the transcripts were calculated for other scion/rootstock combinations subjected to 0 µM or 50 µM CdCl_2_. A gene expression heatmap was generated for the log base 2 average expression fold values using the heatmap.2 () command in the “gplots” package in R (http://www.r-project.org/).

### Statistical Analysis

All data were processed in Statgraphics (STN; St. Louis, MO, USA). All data were tested for normality before the statistical analyses. Three-way ANOVA was applied using CdCl_2_ (Cd), rootstock (R), and scion (S) as the three factors to test all parameters for significant changes. *A posteriori* means comparisons were performed by Tukey’s method when statistically significant interactions were detected. All the results were presented as means ± standard error (SE) of three biological replicates. Differences between means were considered significant at *P* < 0.05 according to the three-way ANOVA *F*-test.

## Results

### Plant Gas Exchange, Chlorophyll Content, and Growth

To explore the Cd-induced phytotoxic effects, gas exchange, photosynthetic pigments, and biomass were determined (**Table 1**, **Figure S1**). There were remarkable differences in gas exchange among the four graft combinations. In the absence of Cd, the CO_2_ assimilation rates and *g_s_* were markedly higher in HF than FJ, regardless of the rootstock (**Table 1**). Relative to their respective controls, the four graft combinations presented with dramatically lower CO_2_ assimilation rates, *g_s_*, and *E* in response to 50 μM Cd exposure for 18 d (**Table 1**). The reductions in CO_2_ assimilation rate, *g_s_*, and *E* were the highest for HF/Mm, but the lowest for HF/Mb (**Table 1**). Cd exposure significantly decreased foliar Chl(a+b) in all four graft combinations. However, the reduction was more pronounced in FJ/Mm than the other three graft combinations (**Table 1**). Cd stress significantly inhibited plant growth (**Table 1**, **Figure S1**). Large differences in scion and rootstock tissue biomass were observed among the various graft combinations (**Table 1**). Root, scion stem, and leaf biomass were always greater in HF than FJ, irrespective of rootstock and Cd conditions (**Table 1**). Cd exposure decreased root biomass in FJ/Mb, rootstock stem and leaf biomass in HF/Mm, and scion stem biomass in all graft combinations (**Table 1**). The inhibitory effects of Cd exposure on scion stem biomass were signiﬁcantly less pronounced in HF and FJ grafted onto Mb than they were for the same scions grafted onto Mm (**Table 1**).

**Table 1:** CO_2_ assimilation rate (*A*, μmol CO_2_ m^−2^ s^−1^), stomatal conductance (*g_s_*, mol H_2_O m^−2^ s^−1^), transpiration rate (*E*, mmol H_2_O m^−2^ s^−1^) and photosynthetic pigments (mg g^−1^ DW) in leaves, and the dry mass (g) of root, rootstock stem, scion stem and leaf tissues of four scion/rootstock combinations exposed either to 0 µM or 50 µM CdCl_2_ for 18 d.

Scion/rootstock combinations	Cd(μM)	*A*	*g_s_*	*E*	chl (a+b)	Root	Rootstock stem	Scion stem	Leaf
HF/Mb	0	13.27 ± 0.18^d^	0.17 ± 0.03^b^	2.97 ± 0.54^b^	6.52 ± 0.22^c^	5.10 ± 0.07^c^	1.58 ± 0.24^ab^	4.17 ± 0.01^e^	3.88 ± 0.29^d^
	50	2.17 ± 0.13^b^	0.02 ± 0.00^a^	0.87 ± 0.12^a^	4.97 ± 0.20^b^	4.71 ± 0.23^bc^	1.48 ± 0.08^a^	3.76 ± 0.02^d^	3.59 ± 0.13^d^
FJ/Mb	0	7.50 ± 0.15^c^	0.16 ± 0.03^b^	3.33 ± 0.37^b^	4.89 ± 0.21^b^	4.29 ± 0.44^b^	1.65 ± 0.01^ab^	2.49 ± 0.11^c^	2.61 ± 0.23^bc^
	50	0.77 ± 0.09^a^	0.02 ± 0.00^a^	0.73 ± 0.13^a^	3.69 ± 0.16^a^	3.35 ± 0.02^a^	1.32 ± 0.03^a^	1.80 ± 0.16^ab^	2.16 ± 0.18^b^
HF/Mm	0	14.37 ± 0.75^e^	0.18 ± 0.01^b^	3.53 ± 0.15^b^	6.49 ± 0.12^c^	5.20 ± 0.20^c^	1.84 ± 0.07^b^	3.64 ± 0.12^d^	5.28 ± 0.07^e^
	50	0.40 ± 0.06^a^	0.01 ± 0.00^a^	0.37 ± 0.03^a^	4.94 ± 0.02^b^	4.78 ± 0.21^bc^	1.41 ± 0.01^a^	2.63 ± 0.09^c^	3.00 ± 0.07^d^
FJ/Mm	0	12.80 ± 0.10^d^	0.16 ± 0.02^b^	3.57 ± 0.45^b^	8.35 ± 0.17^d^	2.79 ± 0.39^a^	1.56 ± 0.16^ab^	2.53 ± 0.21^c^	1.48 ± 0.12^a^
	50	2.20 ± 0.12^b^	0.03 ± 0.00^a^	1.03 ± 0.12^a^	5.23 ± 0.07^b^	2.73 ± 0.10^a^	1.50 ± 0.03^a^	1.52 ± 0.05^a^	1.44 ± 0.01^a^
*P*-values	Cd	****	****	****	****	ns	****	****	****
	S	****	ns	ns	ns	****	*	****	****
	R	****	ns	ns	****	*	*	***	*

Data are presented as the means of three biological replicates ( ± SE). Different letters following the values in the same column indicate significant difference between the treatments. P-values of the ANOVAs of Cd^2+^ (Cd), scion (S) and rootstock (R) are indicated. *P ≤ 0.05, ***P ≤ 0.001, ****P ≤ 0.0001; ns, not significant. HF/Mb and FJ/Mb, “Hanfu” (HF) and “Fuji” (FJ) apple cultivars grafted onto M. baccata (Mb) rootstock, respectively; HF/Mm and FJ/Mm, “Hanfu” (HF) and “Fuji” (FJ) apple cultivars grafted onto M. micromalus “qingzhoulinqin” (Mm) rootstock, respectivelyy. Chl (a + b): sum of chlorophyll a and b.

### Cd Localization, Concentration, BCF, and *T_f_*

**Figure 1:**
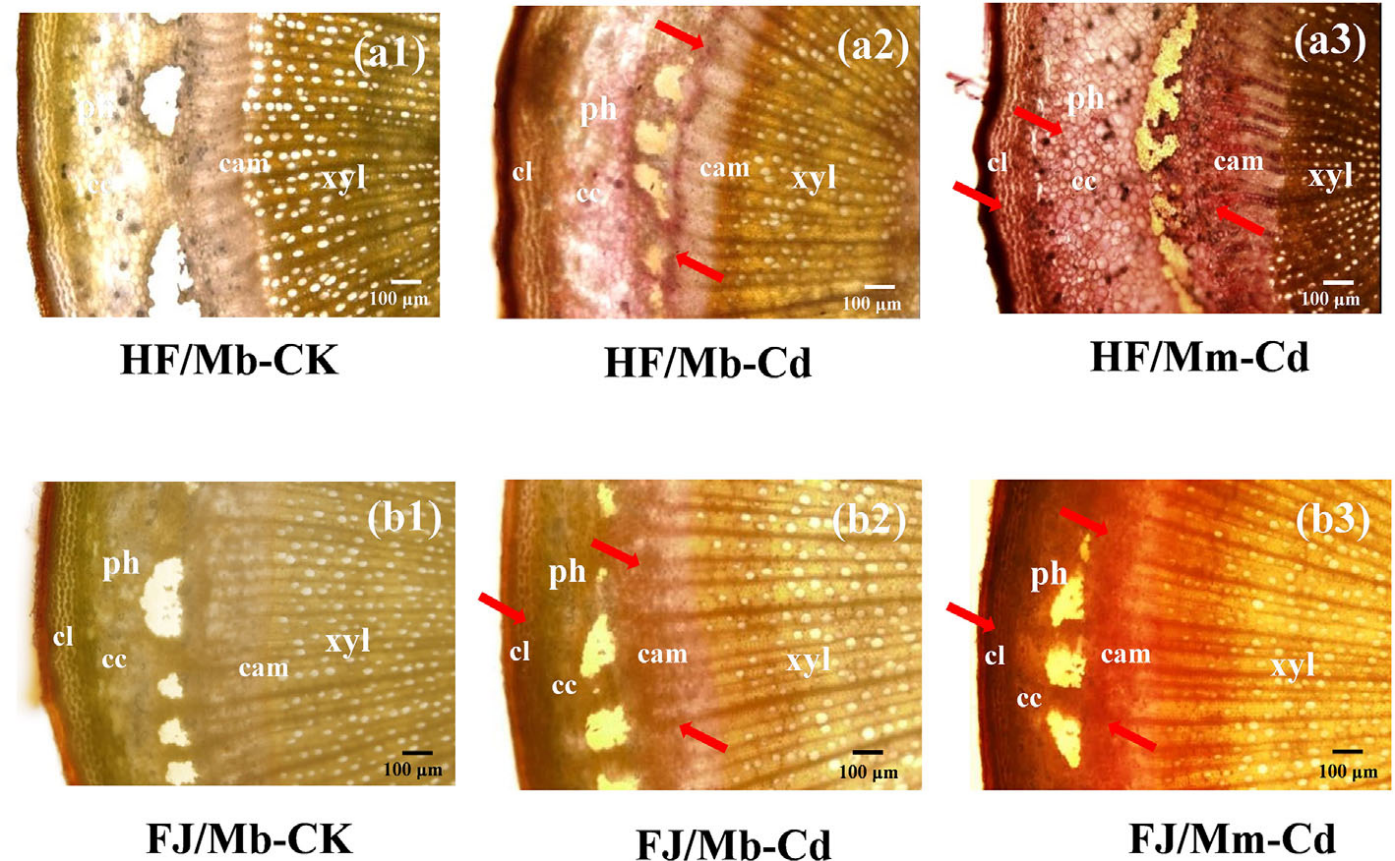
Cadmium (Cd) localization in the scion stems of four scion/rootstock combinations exposed either to 0 µM CdCl_2_ (CK) or 50 µM CdCl_2_ (Cd) for 18 d. The figures shown were representative of three biological replicates. Arrows point to Cd-dithizone precipitates. Histochemical Cd detection in “Hanfu” (HF) and “Fuji” (FJ) scion stems grafted onto *M. micromalus* “qingzhoulinqin” (Mm) under 0 µM CdCl_2_ resembled those for HF and FJ grafted onto *M. baccata* (Mb). HF/Mb and FJ/Mb, “Hanfu” (HF) and “Fuji” (FJ) apple cultivars grafted onto *M. baccata* (Mb) rootstock, respectively; HF/Mm and FJ/Mm, “Hanfu” (HF) and “Fuji” (FJ) apple cultivars grafted onto *M. micromalus* “qingzhoulinqin” (Mm) rootstock, respectively; xyl, xylem; ph, phloem; cam, cambium; cc, cortical cells; cl, collenchyma.

No Cd-dithizone complexes were detected in the roots or scion stems of any scion/rootstock combination not exposed to Cd (**Figures 1** and **S2**). In the roots, Cd was localized mainly to the epidermal cells (**Figure S2**). For the scion stems, Cd was enriched in the phloem collenchyma and cortex (**Figure 1**). The Cd localization pattern was similar for all combinations (**Figures 1** and **S2**). However, Cd staining was more intense in the roots and scion stems of HF and FJ grafted onto Mm than it was in the same organs of HF and FJ grafted onto Mb after Cd treatment (**Figures 1** and **S2**).

**Figure 2:**
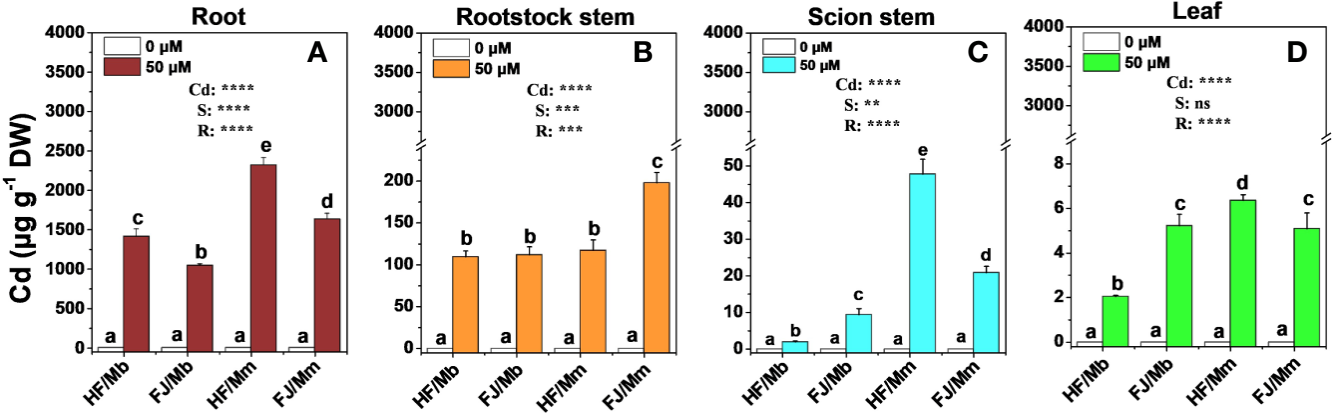
Cd concentrations in the root **(A)**, rootstock stem **(B)**, scion stem **(C)** and leaf **(D)** tissues of four scion/rootstock combinations exposed either to 0 µM or 50 µM CdCl_2_ for 18 d. Data are presented as the means of three biological replicates ( ± SE). Different letters on the bars indicate significant difference between the treatments. *P*-values of the ANOVAs of Cd^2+^ (Cd), scion (S) and rootstock (R) are indicated. **P ≤ 0.01; ***P ≤ 0.001; ****P ≤ 0.0001; ns: not significant. HF/Mb and FJ/Mb, “Hanfu” (HF) and “Fuji” (FJ) apple cultivars grafted onto *M. baccata* (Mb) rootstock, respectively; HF/Mm and FJ/Mm, “Hanfu” (HF) and “Fuji” (FJ) apple cultivars grafted onto *M. micromalus* “qingzhoulinqin” (Mm) rootstock, respectively.

Relative to the no-Cd treatment, Cd exposure induced considerable accumulation in the roots, rootstock stems, scion stem, and leaves of all plants (**Figure 2**). Most of the Cd accumulated in the roots followed by the rootstock stems, scion stems, and leaves. There were substantial differences among the graft combinations in terms of tissue Cd level. Cd concentrations in the tissues of HF and FJ grafted onto Mm were higher than in the tissues of HF and FJ grafted onto Mb (**Figure 2**). For the same rootstock, the root Cd concentrations were always higher in HF than FJ. HF/Mm presented with the highest root Cd levels (**Figure 2A**). In contrast, Cd concentration in the rootstock stems, scion stems, and leaves trends radically differed from those seen in the roots (**Figures 2B–D**). The Cd concentrations were the highest in the FJ/Mm rootstock stems (**Figure 2B**). However, the order of Cd concentration in the treated scion stems was HF/Mm > FJ/Mm > FJ/Mb > HF/Mb, and leaves was HF/Mm > FJ/Mb and FJ/Mm > HF/Mb.

**Figure 3:**
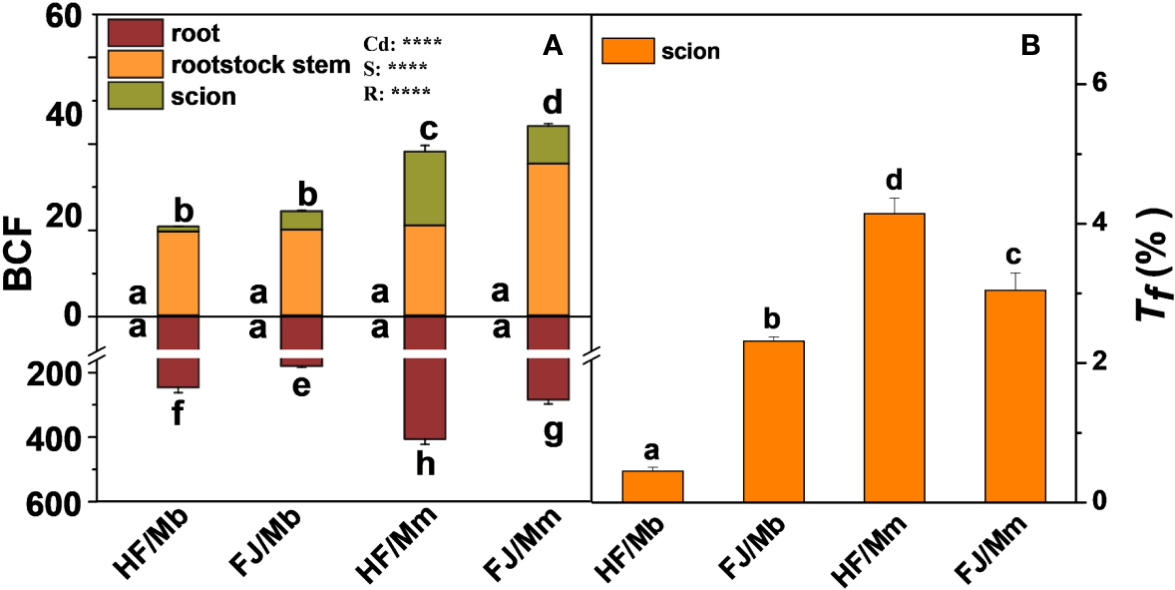
Bio-concentration factor (BCF, **A**) in the roots, rootstock stem and scion, and translocation factor (*T_f_*, **B**) of four scion/rootstock combinations exposed either to 0 µM or 50 µM CdCl_2_ for 18 d. Data are presented as the means of three biological replicates ( ± SE). Different letters on the bars indicate significant difference between the treatments. *P*-values of the ANOVAs of Cd^2+^ (Cd), scion (S) and rootstock (R) are indicated. ****P ≤ 0.0001. HF/Mb and FJ/Mb, “Hanfu” (HF) and “Fuji” (FJ) apple cultivars grafted onto *M. baccata* (Mb) rootstock, respectively; HF/Mm and FJ/Mm, “Hanfu” (HF) and “Fuji” (FJ) apple cultivars grafted onto *M. micromalus* “qingzhoulinqin” (Mm) rootstock, respectively.

The root BCF displayed the same pattern as the root Cd concentration. The rootstock stem and scion BCFs were significantly higher in HF and FJ grafted onto Mm than they were in HF and FJ grafted onto Mb. For the same rootstock, the shoot BCFs were higher in FJ/Mm than they were in HF/Mm. No significant difference in shoot BCF was found between HF/Mb and FJ/Mb (**Figure 3A**). The scion *T_f_* assumed the order HF/Mm > FJ/Mm > FJ/Mb > HF/Mb (**Figure 3B**).

### Subcellular Cd Distribution

Cd was stored mainly in the cell wall fraction (F I) followed by the soluble fraction (F IV), the organelle-rich fraction (F II), and the membrane-containing fraction (F III) of the roots and leaves of the various apple graft combinations (**Table 2**). The Cd concentrations in the foliar and root F I and F IV were strongly influenced by the Cd concentrations in their corresponding tissues. Similar changes in tissue Cd concentration were noted for all graft combinations (**Table 2**). The subcellular Cd levels in F I-IV were always higher for the roots and leaves of HF and FJ grafted onto Mm than they were for the roots and leaves of HF and FJ grafted onto Mb (**Table 2**). However, the pattern of change in the Cd distribution ratios of the four subcellular fractions differed from the change trend in the Cd subcellular concentrations. The Cd distribution ratios for F I were the highest in the roots and leaves of HF/Mb and the lowest in the leaves of FJ/Mm (**Table 2**). The highest Cd distribution ratios for the root and leaf F II were detected in FJ/Mm and HF/Mm while the lowest were detected in the roots and leaf F II of HF/Mb (**Table 2**). The highest and lowest Cd distribution ratios for the root F III were found in FJ/Mm and FJ/Mb, respectively. There were no relative differences in Cd distribution ratio for the leaf F III among the four combinations (**Table 2**). The highest Cd distribution ratios for the root and leaf F IV were measured for HF/Mm and FJ/Mm (**Table 2**).

**Table 2:** Subcellular distribution of Cd and its proportion in root and leaf tissues of four scion/rootstock combinations exposed to 50 µM CdCl_2_ for 18 days.

Tissues	Scion/rootstock	Cd concentration (μg g^−1^ DW)				Cd distribution ratio (%)			
	combinations	F I	F II	F III	F IV	F I	F II	F III	F IV
Root	HF/Mb	664.88 ± 22.33^b^	121.18 ± 2.87^a^	12.55 ± 0.03^b^	480.43 ± 21.08^ab^	51.98 ± 0.55^b^	9.50 ± 0.49^a^	0.98 ± 0.03^c^	37.54 ± 0.80^a^
	FJ/Mb	448.68 ± 17.62^a^	143.41 ± 8.36^a^	4.62 ± 0.45^a^	355.24 ± 18.17^a^	47.12 ± 1.24^a^	15.12 ± 1.18^bc^	0.49 ± 0.05^a^	37.28 ± 1.q7^a^
	HF/Mm	963.13 ± 21.25^c^	257.61 ± 10.09^b^	17.06 ± 1.49^c^	891.16 ± 62.34^c^	45.29 ± 1.69^a^	12.12 ± 0.66^ab^	0.80 ± 0.06^b^	41.79 ± 2.29^b^
	FJ/Mm	660.33 ± 0.58^b^	250.97 ± 17.18^b^	15.37 ± 0.51^c^	537.89 ± 54.95^b^	45.18 ± 1.43^a^	17.20 ± 1.42^c^	1.05 ± 0.06^c^	36.57 ± 2.64^a^
*P*-values	S	*	ns	**	ns	ns	****	ns	ns
	R	****	ns	***	***	ns	**	***	ns
Leaf	HF/Mb	0.90 ± 0.02^a^	0.15 ± 0.01^a^	0.11 ± 0.01^a^	0.64 ± 0.04^a^	50.24 ± 2.22^c^	8.43 ± 0.13^a^	5.82 ± 0.63^a^	35.51 ± 1.47^a^
	FJ/Mb	1.71 ± 0.05^b^	0.83 ± 0.04^b^	0.22 ± 0.01^b^	1.78 ± 0.11^b^	37.69 ± 0.57^b^	18.29 ± 0.80^c^	4.85 ± 0.41^a^	39.16 ± 1.53^a^
	HF/Mm	2.22 ± 0.15^c^	1.19 ± 0.07^c^	0.28 ± 0.03^b^	2.36 ± 0.01^c^	36.62 ± 1.17^b^	19.69 ± 0.36^c^	4.60 ± 0.38^a^	39.09 ± 1.62^a^
	FJ/Mm	1.65 ± 0.07^b^	0.77 ± 0.01^b^	0.24 ± 0.02^b^	2.48 ± 0.07^c^	32.14 ± 0.66^a^	15.11 ± 0.59^b^	4.72 ± 0.35^a^	48.19 ± 0.51^b^
*P*-values	S	****	****	**	****	*	****	ns	ns
	R	Ns	*	ns	****	***	**	ns	**

Data are presented as the means of three biological replicates ( ± SE). Different letters following the values in the same column indicate significant difference between the treatments. P-values of the ANOVAs of scion (S) and rootstock (R) are indicated. *P ≤ 0.05, **P ≤ 0.01, ***P ≤ 0.001, ****P ≤ 0.0001; ns, not significant. HF/Mb and FJ/Mb, “Hanfu” (HF) and “Fuji” (FJ) apple cultivars grafted onto M. baccata (Mb) rootstock, respectively; HF/Mm and FJ/Mm, “Hanfu” (HF) and “Fuji” (FJ) apple cultivars grafted onto M. micromalus “qingzhoulinqin” (Mm) rootstock, respectively. F I, cell wall fraction; F II, organelle-rich fraction; F III, membrane-containing fraction; F IV, soluble fraction.

### O_2_^•−^, H_2_O_2_, MDA, and Non-Enzymatic and Enzymatic Antioxidants

**Figure 4:**
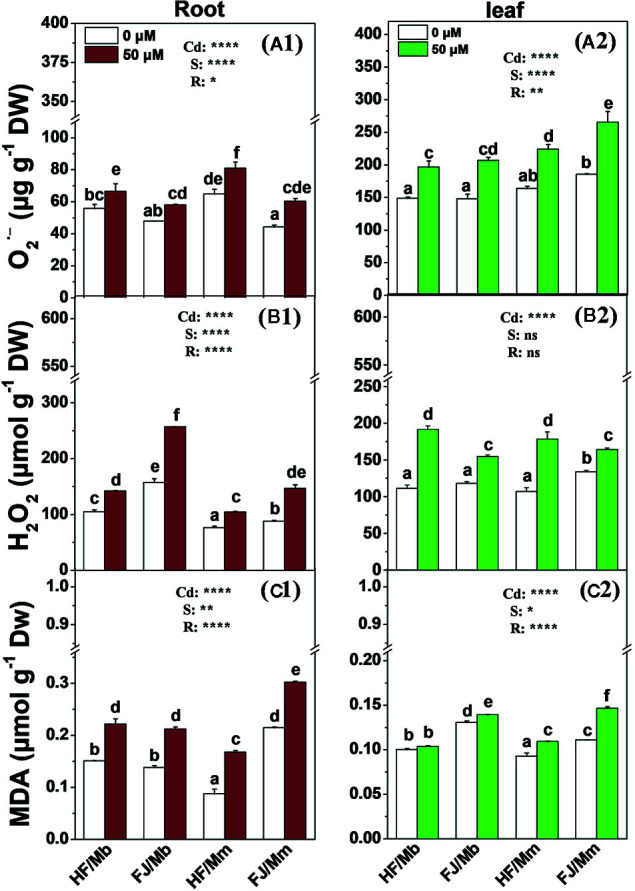
O_2_^.−^ **(A1, A2)**, H_2_O_2_ **(B1, B2)** and MDA **(C1, C2)** in the roots **(A1**–**C1)** and leaves **(A2–C2)** of four scion/rootstock combinations exposed either to 0 µM or 50 µM CdCl_2_ for 18 d. Data are presented as the means of three biological replicates ( ± SE). Different letters on the bars indicate significant difference between the treatments. *P*-values of the ANOVAs of Cd^2+^ (Cd), scion (S) and rootstock (R) are indicated. *P ≤ 0.05; **P ≤ 0.01; ****P ≤ 0.0001; ns: not significant. HF/Mb and FJ/Mb, “Hanfu” (HF) and “Fuji” (FJ) apple cultivars grafted onto *M. baccata* (Mb) rootstock, respectively; HF/Mm and FJ/Mm, “Hanfu” (HF) and “Fuji” (FJ) apple cultivars grafted onto *M. micromalus* “qingzhoulinqin” (Mm) rootstock, respectively.

The root and leaf O_2_^•−^, H_2_O_2_ and MDA concentrations were significantly increased in all four combinations exposed to Cd, relative to their untreated controls, (**Figure 4**). The only exception was the leaf MDA content for HF/Mb (**Figure 4C2**). The rootstock affected the root and leaf O_2_^•−^, H_2_O_2_, and MDA levels with the exception of leaf H_2_O_2_. Cd-induced root and leaf O_2_^•−^, root H_2_O_2_, and leaf MDA were always lower in HF and FJ grafted onto Mb than they were in HF and FJ grafted onto Mm (**Figure 4**). Cd-induced root and leaf O_2_^•−^, root H_2_O_2_, and leaf MDA were always lower in HF than FJ when grafted onto the same rootstocks (**Figure 4**).

**Figure 5:**
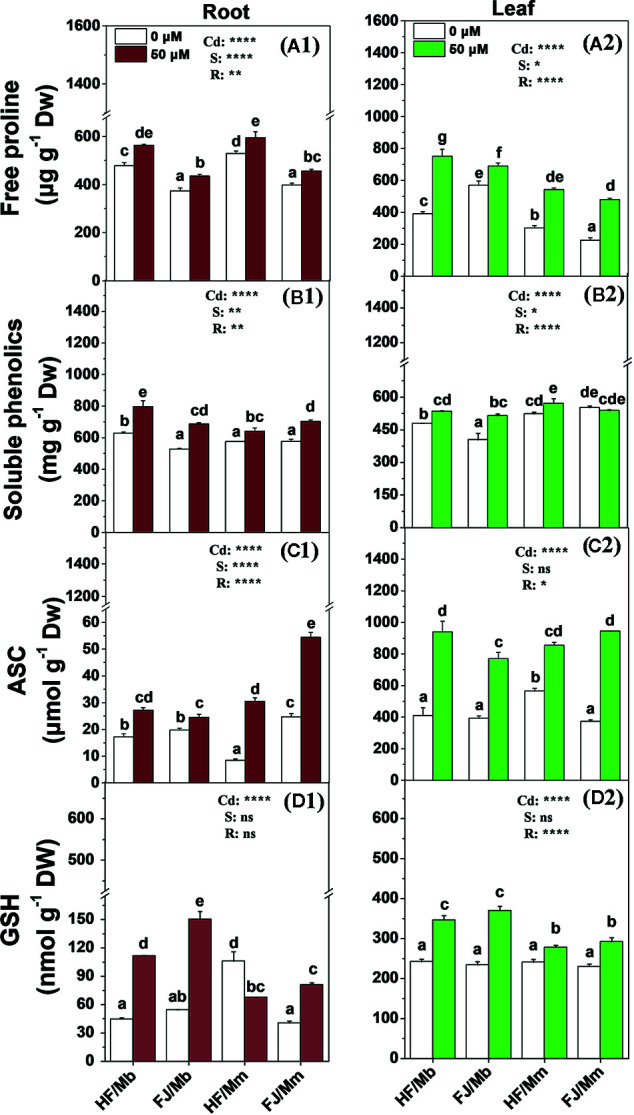
Free proline **(A1**, **A2)**, soluble phenolics **(B1**, **B2)**, ASC **(C1**, **C2)** and GSH **(D1**, **D2)** in the roots **(A1–D1)** and leaves **(A2–D2)** of apple scion/rootstock combinations exposed either to 0 µM or 50 µM CdCl_2_ for 18 d. Data are presented as the means of three biological replicates ( ± SE). Different letters on the bars indicate significant difference between the treatments. *P*-values of the ANOVAs of Cd^2+^ (Cd), scion (S) and rootstock (R) are indicated. *P ≤ 0.05; **P ≤ 0.01; ****P ≤ 0.0001; ns: not significant. HF/Mb and FJ/Mb, “Hanfu” (HF) and “Fuji” (FJ) apple cultivars grafted onto *M. baccata* (Mb) rootstock, respectively; HF/Mm and FJ/Mm, “Hanfu” (HF) and “Fuji” (FJ) apple cultivars grafted onto *M. micromalus* “qingzhoulinqin” (Mm) rootstock, respectively.

Cd exposure significantly induced root and leaf free proline, soluble phenolics, ASC, and GSH production for all four graft combinations (**Figure 5**). The only exceptions were the foliar soluble phenolics for FJ/Mm (**Figure 5B2**) and the root GSH for HF/Mm (**Figure 5D1**). The rootstocks and scions significantly influenced the non-enzymatic antioxidant levels. The Cd-induced increases in foliar free proline and root and leaf soluble phenolics and GSH were more pronounced in HF and FJ grafted onto Mb than they were in HF and FJ grafted onto Mm (**Figure 5**). However, the opposite trend was noted for the root ASC (**Figure 5C1**). The root and leaf free proline, root soluble phenolics, and foliar ASC levels were higher in plants with HF scions grafted onto Mb than in those with FJ scions grafted onto Mb (**Figure 5**).

Unlike the comparatively uniform change profiles of non-enzymatic antioxidants in response to Cd exposure, the changes in the enzymatic antioxidants were complex after Cd treatment (**Figure S3**). Root CAT activity was upregulated only in plants with Mb rootstocks (**Figure S3**). In contrast, foliar CAT activity was downregulated in FJ/Mb, HF/Mm, and FJ/Mm after Cd treatment (**Figure S3**). Cd exposure increased root APX by 206.9%, 66.5%, and 57.7% for HF/Mb, HF/Mm, and FJ/Mm, respectively, and foliar APX by 72.1% for FJ/Mb. However, it inhibited root and leaf APX activity in the other graft combinations (**Figure S3**). Cd treatment inhibited root GR in HF/Mb, FJ/Mb, and HF/Mm but promoted root GR in FJ/Mm (**Figure S3**). Cd exposure reduced foliar GR activity in HF/Mm but had no effect on foliar GR in the other graft combinations (**Figure S3**). In response to Cd exposure, HF grafted onto Mb presented with higher root CAT, APX, and GR activity than FJ grafted onto Mb. However, the opposite results were observed for the foliar activity levels of the aforementioned enzymes in HF and FJ grafted onto Mm (**Figure S3**).

### Transcript Levels of Genes Involved in Cd Transport and Detoxification

**Figure 6:**
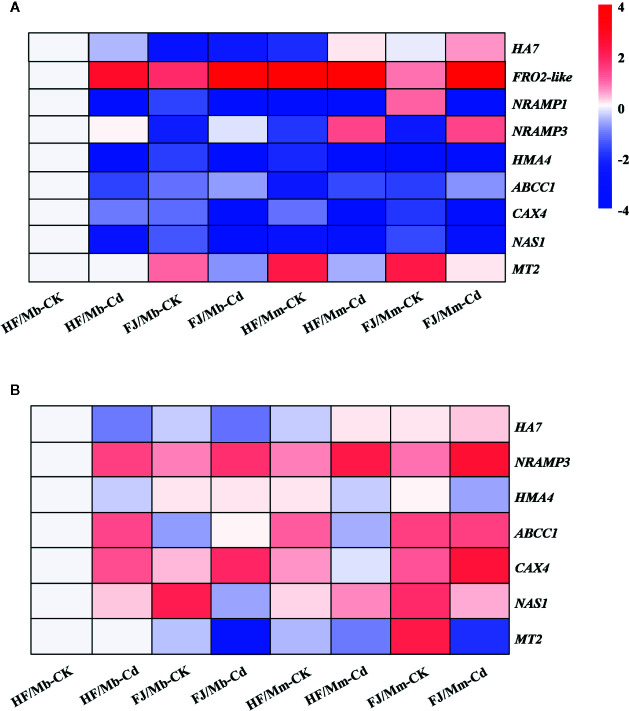
Heatmap of genes encoding proteins involved in Cd uptake, transport and detoxiﬁcation in the roots **(A)** and leaves **(B)** of four scion/rootstock combinations exposed either to 0 µM CdCl_2_ (CK) or 50 µM CdCl_2_ (Cd) for 18 d. For each gene, the expression level was set to 1 in the roots or leaves of “Hanfu” (HF) grafted on *M. baccata* (Mb) rootstock without Cd exposure, and the corresponding fold changes were calculated in other scion/rootstock combinations under 0 or 50 µM CdCl_2_ treatments. The gene expression heatmap was generated on the log base 2 average expression fold values. HF/Mb and FJ/Mb, “Hanfu” (HF) and “Fuji” (FJ) apple cultivars grafted onto *M. baccata* (Mb) rootstock, respectively; HF/Mm and FJ/Mm, “Hanfu” (HF) and “Fuji” (FJ) apple cultivars grafted onto *M. micromalus* “qingzhoulinqin” (Mm) rootstock, respectively.

To elucidate the molecular mechanisms governing the observed morphophysiological changes in the graft combinations in response to Cd stress, we analyzed nine genes putatively implicated in Cd uptake, transport, and detoxification in the roots and leaves (**Figures 6** and **S4**). The *HA7* mRNA levels increased by 4.6-fold and by 1.7-fold in the roots of HF and FJ, respectively, grafted onto Mm when exposed to Cd. On the other hand, they remained unchanged in the FJ/Mb roots and markedly decreased in the HF/Mb roots in response to Cd exposure (**Figure 6A**). *FRO2-like* encoding Fe(III) reductase proteins and *NRAMP1* also regulate Cd uptake (Gao et al., 2011; Lin and Aarts, 2012). Here, the root transcript levels of *FRO2-like* and *NRAMP1* were differentially expressed in response to Cd exposure (**Figure 6A**). Cd treatment upregulated *FRO2-like* but downregulated *NRAMP1* in the roots of all graft combinations (**Figure 6A**). In response to Cd, the *FRO2-like* and *NRAMP1* mRNA levels were always higher in the roots of HF and FJ grafted onto Mm than they were in the roots of HF and FJ grafted onto Mb (**Figure 6A**). The root *HA7*, *FRO2-like*, and *NRAMP1* mRNA levels varied greatly among the four graft combinations, irrespective of rootstock type and Cd exposure (**Figure 6A**). Cd exposure caused a more significant increase in root *FRO2-like* mRNA for FJ than it did for HF, regardless of rootstock type (**Figure 6A**).

NRAMP3 and HMA4 are metal efﬂux transporters, which move Cd from root to shoot. Cd exposure markedly upregulated *NRAMP3* mRNA in all graft combinations except HF/Mb (**Figure 6A**). In contrast, Cd exposure downregulated *HMA4* mRNA in the roots of four graft combinations subjected to Cd (**Figure 6A**). The *NRAMP3* and *HMA4* mRNA levels significantly varied among the four graft combinations, regardless of Cd exposure. The *NRAMP3* and *HMA4* expression levels were lower in the HF and FJ scions grafted onto Mb than they were in the HF and FJ scions grafted onto Mm (**Figure 6A**).

ABCC1, CAX4, MT2, and NAS1 may be involved in plant Cd detoxification. In response to Cd exposure, *ABCC1* was markedly upregulated in the roots of all graft combinations except HF/Mb. In contrast, the *CAX4* and *NAS1* mRNA levels were decreased in the roots of all graft combinations following Cd treatment (**Figure 6A**). After Cd exposure, the apple homolog *MT2* transcript levels were downregulated in the roots of FJ/Mb, HF/Mm, and FJ/Mm compared with their respective controls. However, no change in *MT2* transcript level was detected in the roots of HF/Mb following Cd treatment. The mRNA levels of the aforementioned genes were influenced by the rootstock and scion, (**Figure 6A**). After Cd exposure, the *CAX4* and *MT2*, transcript levels were significantly higher for HF/Mb than they were for the other graft combinations (**Figure 6A**).

In response to Cd treatment, the foliar *HA7* transcript levels were relatively higher for HF/Mm and FJ/Mm but comparatively lower for HF/Mb and FJ/Mb (**Figure 6B**). Cd exposure upregulated *NRAMP3* but downregulated *HMA4* in all graft combinations except FJ/Mb (**Figure 6B**). After Cd exposure, the *NRAMP3* mRNA levels were markedly lower in the scions grafted onto Mb than they were in the scions grafted onto Mm (**Figure 6B**). Moreover, following Cd treatment, the foliar *NRAMP3* transcript levels were significantly lower for HF than they were for FJ, regardless of rootstock type (**Figure 6B**). The Cd treatments increased the *ABCC1* and *CAX4* expression levels in all graft combinations except *ABCC1* in HF/Mm and FJ/Mm and *CAX4* in HF/Mm (**Figure 6B**). Cd exposure caused the strongest upregulation of *ABCC1* and *CAX4* in HF/Mb and FJ/Mb, respectively (**Figure 6B**). The Cd treatments also increased the foliar *NAS1* transcript levels for HF/Mb and HF/Mm but decreased them for FJ/Mb and FJ/Mm (**Figure 6B**). Cd exposure significantly downregulated foliar *MT2* transcription in FJ/Mb, HF/Mm, and FJ/Mm but had no apparent effect on foliar *MT2* transcription in HF/Mb (**Figure 6B**). Following Cd exposure, the foliar *NAS1* and *MT2* transcript levels were always higher for HF than for FJ grafted to the same type of rootstock (**Figure 6B**).

## Discussion

### Cd Tolerance and Accumulation Is Mediated by Both Rootstock and Scion

Reductions in photosynthesis, growth, and photosynthetic pigments are responses of higher plants to Cd toxicity (Luo et al., 2016; Kaya et al., 2019). Grafting plants onto certain rootstocks dramatically alleviates Cd toxicity. Watermelons grafted onto summer squash showed less reduction of shoot and root biomass, chlorophyll content, and photosynthetic efficiency than those grafted onto winter squash (Shirani Bidabadi et al., 2018). Here, the apple cultivar scions HF and FJ grafted onto Mb rootstocks presented with relatively less reduction of CO_2_ assimilation rate, Chl(a+b) concentration, and shoot biomass than HF and FJ grafted onto Mm (**Table 1**). Thus, the Cd tolerance of a graft complex depends on the rootstock genotype. Further, the Mb rootstock efficiently improved Cd tolerance in apple trees. The comparatively higher root, scion stem, and leaf biomass in HF than FJ under Cd exposure indicated that the scion itself also influenced Cd tolerance. This finding was consistent with those reported for *Citrus* in response to Al stress (Liao et al., 2015). Of the four scion/rootstock combinations tested here, HF/Mb exhibited the highest Cd tolerance.

It has been demonstrated that the rootstock affects heavy metal accumulation and translocation to the shoots (Stachowiak et al., 2016). Cd deposition in the root restricts the translocation of the metal to the shoot. This mechanism is an important factor in plant Cd tolerance (Zhou et al., 2017). Here, the rootstock Mb accumulated less Cd and restricted Cd transport to the shoot more effectively than the rootstock Mm (**Figures 1**–**3**). Therefore, Mb may have well-coordinated physiological mechanisms that limit Cd accumulation and translocation and mitigate its deleterious effects. This hypothesis was corroborated by the relatively lower biomass reduction in the scion grafted onto the Mb rootstock (**Table 1**). Numerous studies have attempted to limit Cd translocation to the shoots and enhance Cd tolerance by grafting scions onto special rootstocks (Savvas et al., 2010). However, few investigations have explored the effects of scion cultivars on Cd uptake and accumulation. Zhen et al. (2010) reported that grafting cucumber with salt-tolerant scions limited Na^+^ transport to the leaves and partially improved salt tolerance in the seedlings. In the present study, the type of scion significantly influenced BCF, *T_f_*, and Cd concentration in all tissues (**Figures 2** and **3**). HF grafted onto Mb had lower leaf and stem Cd concentrations than FJ grafted onto the same rootstock. Nevertheless, the opposite results were obtained when the Mm rootstock was used. Therefore, Cd accumulation and translocation also depend on the scion type in *Malus*. The Cd tolerance was greater in HF/Mb than the other three scion/rootstock combinations because the former accumulated and translocated comparatively less Cd. However, there might be a complex communication mechanism within specific scion/rootstock combinations that regulate Cd accumulation and root-to-shoot translocation. The physiological and molecular mechanisms underlying the inhibition of root Cd uptake and acropetal translocation by rootstock–scion interactions remain unclear.

### Rootstock–Scion Interactions Affect Subcellular Cd Distribution and Antioxidant Defense

In plants, subcellular Cd distribution is vital to Cd accumulation, migration, and detoxification (Xin et al., 2013). Cell walls are the first barrier restricting the entrance of metals into cells. The wall material may bind Cd and attenuate organellar damage caused by it (Luo et al., 2016). Our previous study demonstrated that subcellular Cd distribution in various parts of the roots differed among apple rootstocks and resulted in differential tolerance to Cd accumulation (Zhou et al., 2017). In the present study, the roots and leaves of HF and FJ grafted onto Mb had higher cell wall fraction (F I) Cd distribution ratios than those of the same cultivar grafted onto Mm (**Table 2**). Hence, Mb may bind Cd in its cell wall and mitigate organellar Cd toxicity. This theory was confirmed by the comparatively lower Cd distribution ratios in the organelle-rich fraction (F II). On the other hand, subcellular HM distribution also varied with scion type. Ralls apple cultivar scions grafted onto the *M*. *hupehensis* Rehd. rootstock sequestered more Cu in their fibrous root cells than Fuji apple cultivar scions grafted onto the same rootstock. Consequently, the Ralls had a higher Cu tolerance than the Fuji (Wang et al., 2016). Here, the roots of HF grafted onto Mb presented with higher Cd distribution ratios in the cell wall fraction (F I) and lower Cd distribution ratios in the organelle-rich fraction (F II) than the roots of FJ grafted onto Mb. Therefore, the scion type also affects subcellular root Cd distribution. As Cd has lower root mobility in HF/Mb than in FJ/Mb, the former may have a relatively higher Cd^2+^ detoxification capacity. Of the four scion/rootstock combinations tested in this study, the roots and leaves of HF/Mb had the highest Cd distribution ratios in their cell wall fraction (F I) but the lowest Cd distribution ratios in their organelle-rich fraction (F II). As a result, HF/Mb had the lowest Cd accumulation and translocation capacity and, by extension, the highest Cd tolerance and the strongest ability to prevent Cd from interfering with the organelles.

Cd is non-essential and toxic to most organisms (Luo et al., 2016). The observed increases in O_2_^•−^, H_2_O_2_, and MDA in the roots and leaves of all apple scion/rootstock combinations exposed to Cd indicated the presence of Cd phytotoxicity. However, the changes in ROS and MDA concentration in response to the Cd treatment differed among the four scion/rootstock combinations. There was less pronounced induction of O_2_^•−^ and MDA in the roots and leaves and H_2_O_2_ in the roots of the apple cultivar scions grafted onto Mb than there was in those grafted onto Mm. Therefore, Mb more effectively alleviated Cd toxicity than Mm. The tomato cultivar “Ikram” grafted onto “Maxifort” had lower ROS and MDA levels than self-grafted “Ikram” (Kumar et al., 2015). The scion also significantly affected the root and leaf ROS and MDA concentrations but not the leaf H_2_O_2_ concentrations. Here, we found lower Cd-induced root and leaf MDA and O_2_^•−^ and root H_2_O_2_ production in HF than FJ when they were grafted onto the same rootstock. Hence, HF might partially ameliorate Cd toxicity in apple trees. Zhen et al. (2010) reported that salt stress induced more H_2_O_2_ in the roots of grafted cucumber Jinchun No. 2 (salt-tolerant) than it did in the roots of grafted cucumber Jinyu No. 1 (salt-sensitive) when Figleaf Gourd was the rootstock. Overall, both rootstock and scion control the tissue ROS and MDA concentrations in plants subjected to Cd stress. Thus, the rootstock–scion interaction might regulate the antioxidant system.

To contend with Cd toxicity, plants require non-enzymatic and enzymatic antioxidant mechanisms that effectively scavenge Cd-induced ROS (He et al., 2015). Non-enzymatic antioxidants such as free proline, soluble phenolics, ASC, and GSH in the roots and leaves and enzymatic antioxidants including CAT and APX in the roots may play key roles in Cd detoxification (Anjum et al., 2016; Guo et al., 2019). However, the significant differences in antioxidant level in response to Cd stress among the various apple scion/rootstock combinations suggest that the stress defense pathways are influenced by both the rootstock and the scion. The elevated concentrations of free proline in the leaves, soluble phenolics in the roots, and GSH in the roots and leaves of apple cultivars grafted onto Mb were associated with the lower O_2_^•−^ and MDA concentrations observed in the same scion/rootstock combination. These results suggest that all apple scion genotypes have relatively higher capacities to scavenge Cd-induced ROS when they are grafted onto Cd-tolerant rootstocks such as Mb rather than Cd-sensitive ones such as Mm. Similar findings were reported for *Citrus* (Hippler et al., 2016), watermelon (Shirani Bidabadi et al., 2018), and *Cyphomandra betacea* (Liu et al., 2019) under HM stress. Although a great deal of work has been done to improve the crop antioxidant system under Cd stress by grafting the scions onto Cd-tolerant rootstock, few studies have evaluated the effects of grafting with different scion genotypes. In the present study, the influences of the scion on free proline, soluble phenolics, and root ASC and enzyme activity (except for APX) were all significant. For this reason, the scion plays important roles in enhancing antioxidant capacity and alleviating Cd-induced oxidative damage. In response to Cd exposure conditions, the free proline, soluble phenolics, and ASC concentrations were higher in the roots of HF grafted onto Mb than they were in the roots of FJ grafted onto Mb. Hence, HF more efficiently ameliorated Cd toxicity than FJ when it was grafted onto the Mb rootstock.

### Expression Levels of Genes Involved in Cd Uptake, Translocation, and Tolerance Were Affected by Rootstock and Scion

The four apple scion/rootstock combinations differed in terms of Cd accumulation and tolerance because of the influences of both the rootstock and the scion. It was expected that scion/rootstock interactions affected the transcription of the genes involved in Cd uptake, translocation, and detoxification. Plasma membrane-bound H^+^-ATPases, Fe(III) reductase, and NRAMPs regulate the uptake of divalent cations such as Cd^2+^ in plants (Zhou et al., 2016; Ding et al., 2017; He et al., 2020). *HA7* and *FRO2-like* encode PM-bound H^+^-ATPase and Fe(III) reductase, respectively. *HA7*, *FRO2-like*, and *NRAMP1* downregulation in *M. baccata* roots lowered net Cd^2+^ influx in this species more than it did in other apple rootstocks (Zhou et al., 2016). Here, the root *HA7*, *FRO2-like*, and *NRAMP1* mRNA levels and the leaf *HA7* mRNA levels were lower in Cd-challenged HF and FJ grafted onto Mb than they were in Cd-challenged HF and FJ grafted onto Mm This observation was consistent with the fact that the Cd concentrations in the roots and leaves of these scions were relatively lower when Mb was the rootstock. Therefore, Mb might effectively prevent foliar Cd uptake because the rootstock affects transcription of the genes controlling Cd uptake. Si et al. (2010) reported that the rootstock significantly affected gene expression in the *Citrullus colocynthis* scion, and certain signals transported from the root to the shoot may influence Cd uptake. The significant differences in the root *HA7*, *FRO2-like*, and *NRAMP1* mRNA levels between scions grafted onto the same rootstock indicated that the scion genotype also influenced the transcription of the genes involved in Cd uptake. There were relatively higher *HA7* and *NRAMP1* mRNA levels in the roots of Cd-treated HF/Mb than in those of Cd-treated FJ/Mb. This discovery corresponded well to the comparatively higher Cd accumulation measured in the roots of HF/Mb than in those of FJ/Mb.

Tonoplast-localized *NRAMP3* and PM-localized *HMA4* play key roles in plant Cd transport. *AtNRAMP3* is required for HM vacuolar remobilization and root-to-shoot translocation (Thomine et al., 2000; Oomen et al., 2009). *AtHMA4* overexpression enhanced root-to-shoot Cd translocation (Verret et al., 2004). The *NRAMP3* and *HMA4* transcript levels were altered in apple rootstocks subjected to Cd. Therefore, these genes probably participate in Cd translocation in *Malus* (Zhou et al., 2016). In this study, the observed *HMA4* downregulation in all scion/rootstock combinations suggested that these plants limit Cd translocation to the shoots and alleviate Cd toxicity and injury to the photosynthetic apparatus. Compared to the rootstock Mm, Mb more effectively restricted leaf Cd accumulation and translocation in the scion. This may be related to the fact that the downregulation of *NRAMP3* and *HMA4* mRNA was greater in Mb than in Mm. Moreover, compared to FJ/Mb, the comparatively greater repression of *HMA4* transcription and unaltered *NRAMP3* expression in HF/Mb roots subjected to Cd resulted in relatively less Cd transport to the HF/Mb shoots. These results suggest that the scion also affects transcription of the genes involved in Cd transport. Further, HF downregulated these genes when it was grafted onto Mb.

To alleviate Cd phytotoxicity, cytosolic Cd may be chelated by NA, MT, and PC, and the Cd cations and PC–Cd chelates may be sequestered into vacuoles by CAX4 and ABCC1 (Mei et al., 2009; Park et al., 2012; Luo et al., 2016). Transgenic *Arabidopsis* overexpressing *CAX4* presented with elevated vacuolar Cd^2+^ sequestration and, by extension, higher Cd tolerance than the wild type (Mei et al., 2009). Our previous study found that the mRNA levels of *ABCC1* and *NAS1* catalyzing NA synthesis were higher in *M. baccata* than in *M. micromalus* “qingzhoulinqin”. Hence, the former had relatively greater Cd tolerance (He et al., 2020). The *ABCC1*, *CAX4*, and *MT2* transcript levels were substantially higher in Cd-treated HF/Mb than they were in Cd-challenged HF/Mm which led to comparatively higher Cd tolerance in the former. This theory was confirmed by the relatively less ROS and MDA accumulation and enhanced antioxidant defense in HF/Mb. The expression patterns of the genes involved in Cd detoxification markedly differed between HF and FJ grafted onto Mb and Mm. The communication mechanisms among rootstocks and scions might be highly complex and diverse. Thus, gene expression in response to Cd stress must be assessed for each scion/rootstock combination rather than each rootstock or scion alone. Differential transcriptional regulation of *ABCC1*, *CAX4*, *MT2*, and *NAS1* in various scions grafted onto the same rootstock indicated that the scions also modulated the mRNA levels of the genes involved in Cd detoxification. Certain signals may participate in root-to-shoot Cd transport and influence the transcriptional regulation of the genes involved in Cd uptake, translocation, and detoxification (Savvas et al., 2010). Therefore, further research is required to elucidate the scion-rootstock interaction regulating Cd uptake, translocation, and detoxification and the changes in putative signaling factors such as calcium ion and phytohormones in *Malus*.

## Conclusion

Cd accumulation and tolerance in *Malus* plants depends on the rootstock, scion, and rootstock–scion interaction. HF and FJ apple scions grafted onto Mb rootstocks alleviated Cd-induced decreases in photosynthetic rate, photosynthetic pigment, and biomass. Mb may have restricted Cd accumulation and translocation to the shoot. The comparatively higher Cd tolerance in HF and FJ grafted onto Mb than those grafted onto Mm may be attributed to the fact that the root cell walls of Mb immobilized Cd and reduced O_2_^•−^ and MDA foliar accumulation. Further, the scions grafted onto Mb presented with relatively higher foliar free proline, ASC and GSH concentrations and CAT activity. Thus, grafting apple scions onto the Cd-tolerant Mb rootstock increased the apple seedling antioxidant capacity. Scion genotype also affects the photosynthetic rate, biomass, Cd accumulation and translocation, and ROS and antioxidant levels. Compared with FJ exposed to Cd, HF presented with lower foliar O_2_^•−^, root H_2_O_2_, and root and leaf MDA concentrations but higher ROS-scavenging capacity when grafted onto Mb. In addition to regulating physiological acclimation, the rootstock, scion, and the rootstock–scion interaction influenced the mRNA transcript levels of several genes participating in Cd uptake, transport, and detoxification including *HA7*, *FRO2-like*, *NRAMP1*, *NRAMP3*, *HMA4*, *MT2*, *NAS1*, and *ABCC1*. Therefore, the responses of grafted *Malus* plants to Cd toxicity vary with rootstock, scion, and rootstock–scion interaction. Therefore, each scion/rootstock combination rather than each separate rootstock and scion must be assessed for Cd accumulation and tolerance.

## Data Availability Statement

All datasets presented in this study are included in the article/**Supplementary Material**.

## Author Contributions

JH and SQ conceived and designed research. JH, JZ, HW, and XZ performed the experiment. JH, JZ, SQ, and DL analyzed data and prepared the manuscript. HL provided plant materials. All authors contributed to the article and approved the submitted version.

## Funding 

This work was supported by the National Natural Science Foundation of China (Grant No. 31501712, 31972359), the Liaoning Revitalization Talents Program (Grant No. XLYC1907105), the Scientific Research Foundation of Liaoning (Grant No. LSNQN201916), the National Key Research and Development Program of China (Grant No. 2016YFD0201115), the Program for Liaoning Excellent Talents in University (Grant No. LJQ2015098), the China Agriculture Research System (Grant No. CARS-27), and the Scientiﬁc Research Foundation of Talent Introduction of Shenyang Agricultural University (Grant No. 20153007).

## Conflict of Interest

The authors declare that the research was conducted in the absence of any commercial or financial relationships that could be construed as a potential conflict of interest.
